# Role of Laser Iridoplasty in the Management of Angle Closure Mechanisms other than Pupillary Block

**DOI:** 10.5005/jp-journals-10008-1166

**Published:** 2014-06-12

**Authors:** Vitor Gomes Prado, Syril Dorairaj, Luis Gustavo Biteli, Aline KS Sousa, Pilar AM Moreno, Flavio Siqueira Lopes, Tiago Santos Prata

**Affiliations:** Resident, Department of Ophthalmology, Glaucoma Services, Fiederal University of São Paulo, São Paulo, Brazil; Associate Professor, Department of Ophthalmology, Glaucoma Anterior Segment Surgery, Mayo Clinic, Jacksonville, Florida, USA; Medical Staff, Department of Ophthalmology, Glaucoma Services, Fiederal University of São Paulo, Hospital Medicina dos Olhos, São Paulo, Brazil; Medical Staff, Department of Ophthalmology, Glaucoma Services, Fiederal University of São Paulo, São Paulo, Brazil; Medical Staff, Department of Ophthalmology, Glaucoma Services, Fiederal University of São Paulo, Hospital Medicina dos Olhos, São Paulo, Brazil; Glaucoma Fellow, Department of Ophthalmology, Glaucoma Services, Fiederal University of São Paulo, Hospital Medicina dos Olhos, São Paulo, Brazil; Associate Professor, Department of Ophthalmology, Glaucoma Services, Fiederal University of São Paulo, Hospital Medicina dos Olhos, São Paulo, Brazil

**Keywords:** Angle closure, Nonpupillary block mechanisms, Plateau iris, Laser peripheral iridoplasty.

## Abstract

**Purpose:** To investigate the treatment outcomes of argon laser peripheral iridoplasty (ALPI) in angle closure mechanisms other than pupillary block.

**Methods:** We conducted a comprehensive chart review to evaluate consecutive patients who underwent ALPI due to unsuccessful laser iridotomy (whenever the angles remained occludable) between July 2009 and April-2012. An occludable angle was defined as the posterior trabecular meshwork not visible for ≤180° without indentation on dark room gonioscopy. Eyes with previous incisional surgery or more than 90° of peripheral anterior synechiae were excluded. Main data collected were age, presence of glaucoma, pre- and postlaser intraocular pressure (IOP), angle-status, and underlying angle closure mechanism. Main outcomes were post ALPI angle widening on gonioscopy and magnitude of IOP reduction.

**Results:** A total of 41 eyes (27 patients) with persistent occlu-dable angles were initially included in the analysis, comprising approximately 14% of the 196 patients (321 eyes) that had under gone laser iridotomy during the predefined period. Among these cases, most common angle closure mechanisms were plateauiris (56%) and lens-induced component (34%). Patients with plateau iris were mostly women and younger than those with lens-induced component (p ≤ 0.03). A total of 35 eyes (23 patients) underwent ALPI (63% had glaucoma). Mean IOP was significantly reduced from 18. 2 ± 4.7 to 14.6 ± 3.8 (p < 0.01), with no significant difference between patients with plateau iris and lens-induced components (p = 0.22). Over 91% of these eyes showed nonoccludable angles following ALPI (follow-up of 11.8 ± 3.3 months).

**Conclusion:** In this series of middle-aged patients with occlu-dable angles, despite a patent iridotomy, ALPI was a useful procedure independent of the underlying mechanism, leading to angle widening and moderate IOP reduction in most cases.

**How to cite this article:** Prado VG, Dorairaj S, Biteli LG, Sousa AKS, Moreno PAM, Lopes FS, Prata TS. Role of Laser Iridoplasty in the Management of Angle Closure Mechanisms other than Pupillary Block. J Curr Glaucoma Pract 2014;8(2):82-84.

## INTRODUCTION

Relative pupillary block is considered the primary mechanism for angle-closure.^1-3^ Although laser peripheral irido-tomy (LPI) remains the cornerstone of angle closure management, it does not widen the angle in all cases, as approxi mately 20 to 30% of these patients continue to have appositional angle closure in the presence of a patent iridotomy.^1-3^ In these cases, nonpupillary block mechanisms, such as lens-induced, plateau iris and peripheral angle crowding, may be involved.^4-7^ Argon laser peripheral iridoplasty (ALPI) and lens extraction have been suggested as effective procedures to manage eyes with persistent occludable angle.^8-10^

Looking carefully at previously published data, most studies have focused on ALPI effects exclusively in eyes with plateau iris component^9^ or have not specifically defined the underlying angle closure mechanism. In this study, we aimed to investigate the efficacy of ALPI in consecutive eyes with occludable angles despite a patent iridotomy, independent of the underlying angle closure mechanism.

## METHODS

After Institutional Review Board approval, we conducted a comprehensive chart review to evaluate consecutive patients who underwent ALPI due to occludable angles between July 2009 and April 2012. An occludable angle was defined as the posterior trabecular meshwork not visible for ≤180° without indentation on dark room gonioscopy. We included in the analysis all cases that required ALPI due to unsuccessful laser iridotomy (whenever the angles remained occludable). Eyes with previous incisional surgery, with more than 90° of peripheral anterior synechiae, ocular trauma, uveitis, or any other form of secondary glaucoma were excluded. Data collected were: age, gender, race, presence of glaucoma, pre- and postlaser intraocular pressure (IOP), number of antiglaucoma medications, angle-status, and under lying angle closure mechanism as determined by gonioscopy (ultrasound biomicroscopy was used whenever necessary), and subsequent management during follow-up. On gonioscopy, eyes considered to have lens-induced component were those in which the lens extended nearly to the angle wall, so that indentation produced a narrow, inverted triangular space and the iris contour remained smoothly domed into its insertion point. Eyes in which the ‘double hump' sign was identified during indentation were considered to have plateau iris component. Main outcomes were post-ALPI angle widening on gonioscopy and magnitude of IOP reduction.

## RESULTS

A total of 41 eyes (27 patients) with persistent occludable angles despite a patent LPI were initially included in the analysis, comprising approximately 14% of the 196 patients (321 eyes) that had previously undergone iridotomy during the predetermined period. Among these 41 cases, most common angle closure mechanisms were plateau iris (56%) and lens-induced component (34%). Patients with plateau iris were mostly women (75%) and younger (mean age, 55.3 years) than those with lens-induced component (mean age, 64.8 years; 30% were women; p ≤ 0.03). Table 1 provides clinical characteristics of these patients.

Most of these cases of persistent occludable angles, following LPI, were treated with ALPI (85%; 23 patients – 35 eyes). Overall, glaucoma was found in 63% of the eyes undergoing iridoplasty. The average number of preoperative medications was 1.5 ± 1.1. Treatment regimen was not changed until the last follow-up visit. IOP was significantly reduced from 18.2 ± 4.7 to 14.6 ± 3.8 (p < 0.01), with no significant difference between patients with plateau iris and lens-induced components (p = 0.22; follow-up of 11.8 ± 3.3 months). Over 91% of the eyes showed nonoccludable angles following ALPI. In one case, filtering surgery was required to achieve adequate IOP control.

**Table 1:** **Table 1:** Characteristics of patients with persistent occludable angles following laser peripheral iridotomy*

*Variables*		*Patients (n 27; 41 eyes)*	
Mean age (years)		58.9 ± 11.8	
Gender (%) (men/women)		44/56	
Race (%) (White/Black/Asian descendant)		78/15/7	
Presence of glaucoma (% of eyes)		68% (28 out of 41 eyes)	
Angle-closure mechanisms(%)		56/34/10	
(PIC/LIAC/others)			
Mean IOP before ALPI (mm Hg)		18.2 ± 4.7	
Mean IOP after ALPI (mm Hg)		14.6 ± 3.8	

*Data are given as mean ± standard deviation whenever indicated PIS: Plateau iris component; LIAC: Lens-induced angle-closure; IOP: Intraocular pressure; ALPI: Argon laser peripheral iridoplasty

## DISCUSSION

While dealing with cases of angle closure (with or without glaucoma), identification of the underlying mechanism is of utmost importance as each may have a different course and require a different treatment approach. Although there is extensive information in the literature regarding the main etiologies and management guidelines of primary angle closure in several populations, there are scant data when it comes to South American patients (Brazilians, more specifically).^1,11-16^ Evaluating a large series of patients with primary angle closure (with and without glaucoma), we determined the most frequent underlying mechanisms and the outcomes of laser treatment. To the best of our knowledge, this is the first study to report on such findings in a large sample of South American patients.

We believe it is important to discuss the clinical implications of our findings. Primary angle closure glaucoma is a leading cause of bilateral blindness worldwide.^16^ Treat ment of primary angle closure is directed toward two goals: eliminate the mechanism of angle closure and control any remaining IOP elevation.^14^ Although laser irido tomy is currently the first line of treatment, many eyes will continue to have appositional angle closure and an additional treatment option might be necessary.^1,3^ In this large consecutive series, ALPI was highly effective in eliminating residual appositional angle closure caused by mechanisms other than pupillary block. In addition, we documented a 20% IOP reduction on average. Our results not only confirm the important role of ALPI in cases of plateau iris,^9^ but also suggest it as an effective alternative in cases of lens-induced component. We believe this is clinically meaningful, as not every case of lens-induced angle closure coexists necessarily with a symptomatic cataract. This seems to be the case with our study population, as its mean age was under 60 years (mean age 58.3 ± 11.6 years considering the 196 patients). It is noteworthy that ALPI has also been successfully used as an initial treatment to break acute phacomorphic attacks.^17^ As additional findings, our plateau iris patients tended to be women and younger than those with lens-induced component; ALPI effectiveness being similar in both angle closure mechanisms.

## CONCLUSION

Our findings suggest that a significant proportion of patients with angle closure were not completely treated with LPI. In these large series of middle-aged patients with angle closure originating mainly at an anatomic level posterior to the iris, ALPI was a useful procedure independent of the underlying mecha nism, leading to angle widening and moderate IOP reduction in most cases. A detailed eye exam with indentation gonioscopy should always be performed after LPI to rule out persistent angle closure due to nonpupillary block mechanisms.
